# Host Feeding Patterns of *Culex* Mosquitoes and West Nile Virus Transmission, Northeastern United States

**DOI:** 10.3201/eid1203.051004

**Published:** 2006-03

**Authors:** Goudarz Molaei, Theodore G. Andreadis, Philip M. Armstrong, John F. Anderson, Charles R. Vossbrinck

**Affiliations:** * The Connecticut Agricultural Experiment Station, New Haven, Connecticut, USA

**Keywords:** Blood feeding pattern, Mosquitoes, Culex pipiens, Culex restuans, Culex salinarius, Bridge vector, Turdus migratorius, Odocoileus virginianus, West Nile virus, Cytochrome b gene

## Abstract

To evaluate the role of *Culex* mosquitoes as enzootic and epidemic vectors for WNV, we identified the source of vertebrate blood by polymerase chain reaction amplification and sequencing portions of the cytochrome b gene of mitochondrial DNA. All *Cx. restuans* and 93% of *Cx. pipiens* acquired blood from avian hosts; *Cx. salinarius* fed frequently on both mammals (53%) and birds (36%). Mixed-blood meals were detected in 11% and 4% of *Cx. salinarius* and *Cx. pipiens*, respectively. American robin was the most common source of vertebrate blood for *Cx. pipiens* (38%) and *Cx. restuans* (37%). American crow represented <1% of the blood meals in *Cx. pipiens* and none in *Cx. restuans*. Human-derived blood meals were identified from 2 *Cx. salinarius* and 1 *Cx. pipiens*. Results suggest that *Cx. salinarius* is an important bridge vector to humans, while *Cx. pipiens* and *Cx. restuans* are more efficient enzootic vectors in the northeastern United States.

## Abstract 2

*Culex salinarius* is a bridge vector to humans, while *Cx. pipiens* and *Cx. restuans* are more efficient enzootic vectors.

West Nile virus (WNV) has become firmly established in the Western Hemisphere since its discovery in the New York City area in 1999 (*1**,**2*). The virus has spread at an unprecedented rate throughout the continental United States and to neighboring countries, where it is maintained in an enzootic cycle that involves wild birds and ornithophilic mosquitoes (*3*). To date, 60 mosquito species have been found to be infected with WNV in North America; certain *Culex* spp. appear to be primary vectors, depending on region (*4*). In the northeastern United States, *Culex pipiens*, *Cx. restuans*, and *Cx. salinarius* have been implicated as the principal vectors because they are physiologically competent (*5*), frequently infected with the virus in nature, and closely associated with WNV transmission foci (*6*). However, the precise role that each of these species plays in enzootic transmission among birds or epidemic transmission to humans is not entirely clear.

Entomologic measures of risk may be estimated for different mosquito species by considering their abundance, biting behavior, prevalence of WNV infection, and vector competence. By synthesizing these parameters, Kilpatrick et al. (*7*) estimated that *Cx. pipiens* and *Cx. restuans* were responsible for up to 80% of human infections in New York, whereas *Cx. salinarius* accounted for only 4% of such infections. However, in Connecticut, the abundance of *Cx. salinarius* and prevalence of WNV infection in this species often approach those of *Cx. pipiens* (*6*). Observations in rural and urban sites in New York further indicate that *Cx. pipiens* and *Cx. restuans* are largely ornithophilic, whereas *Cx. salinarius* feeds more frequently on mammals (*8*), which supports the idea of a "bridge vector" role for this species. Nevertheless, collections from New Jersey indicate that mosquitoes of the *Cx. pipiens* complex may readily feed on mammals, including humans (*9*). Further blood meal analysis is required from mosquitoes collected in those habitats that support intense WNV transmission to more fully understand their respective roles as enzootic and epidemic vectors. Such information is vital to the success of any vector control program.

The current research initiative was undertaken to characterize the host-feeding patterns of *Culex* vectors and to evaluate their contribution to enzootic maintenance of WNV in wild bird populations and epidemic transmission to humans. Accordingly, blood-fed mosquitoes were collected from WNV transmission foci in Connecticut and analyzed for host source by sequencing polymerase chain reaction (PCR) amplification products of the vertebrate cytochrome b gene.

## Materials and Methods

### Mosquito Collection

Mosquitoes were collected from 31 different sites in 6 counties in Connecticut during a 3-year period (June through October, 2002–2004) as part of a statewide surveillance program (*6*) and a focused trapping effort in Fairfield County (*10*). Most (71%) of the mosquito collection sites were located in densely populated residential locales along the urban/suburban corridor that extends from lower Fairfield and New Haven Counties, where high levels of WNV activity were recorded (Figure, Table 1). Trap sites included parks, greenways, golf courses, undeveloped wood lots, sewage treatment plants, dumping stations, and temporary wetlands associated with waterways. Three trap types were used: a CO_2_-baited CDC light trap (John W. Hock Co., Gainesville, FL, USA), a mosquito magnet experimental trap (American Biophysics Corp., East Greenwich, RI, USA), and a CDC gravid mosquito trap (*11*). Typically, traps were operated overnight and retrieved the following morning. Live, adult mosquitoes were transported to the laboratory, where they were promptly identified on chill tables with a stereomicroscope by using descriptive keys (*12*). All mosquitoes with fresh or visible blood remnants were transferred into individual 2-mL tubes labeled according to species, date of collection, and locale and stored at –80°C.

**Figure:**
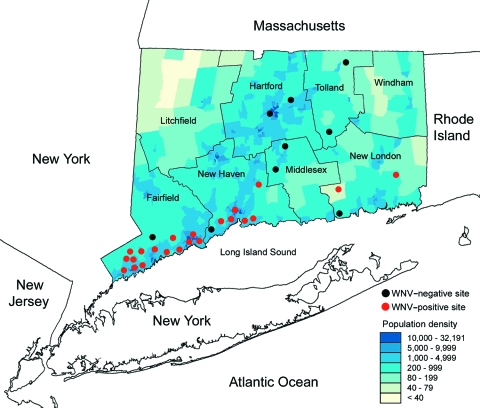
Geographic distribution of West Nile virus isolations from mosquitoes in relation to human population density and mosquito trapping in Connecticut, 2002–2004. "WNV-positive site" indicates that virus isolations were made from mosquitoes collected from these trapping locations.

**Table 1:** No. *Culex* mosquitoes collected for blood-meal analysis from 6 counties in Connecticut, 2002–2004

County	Human population density (per mi^2^)	*Culex pipiens*	*Cx. restuans*	*Cx. salinarius*
Fairfield	1,410	195	25	51
New Haven	1,361	17	5	51
Hartford	1,166	–	1	–
Middlesex	420	–	–	2
New London	389	1	1	2
Tolland	332	–	1	–
Total	–	213	33	106

### DNA Isolation from Blood-fed Mosquitoes

Mosquito abdomens were removed and reserved for blood-meal analysis with the aid of a dissecting microscope. Each mosquito was dissected individually on a new microscope slide by using flame-sterilized forceps to avoid cross-contamination. DNA was isolated from the abdominal contents of blood-fed mosquitoes individually by using DNA-zol BD, (Molecular Research Center, Cincinnati, OH, USA) according to the manufacturer's recommendation. Briefly, individual mosquito abdomens were homogenized with heat-sealed pipette tips in 1.5-mL tubes containing DNA-zol BD solution. The homogenates were incubated at room temperature for 5–10 min, mixed, and then centrifuged at 10,000 × *g* for 10 min. DNA was precipitated by adding isopropanol and 3–4 μL Poly Acryl Carrier (Molecular Research Center). The DNA pellet was then washed twice with 75% ethanol, air-dried briefly, reconstituted in TE buffer (10 mmol/L Tris-HCl, pH 8.0, 1 mmol/L EDTA) and stored at –20°C for further analysis.

### Blood-meal Analysis

Isolated DNA from the mosquito blood meals served as DNA templates in subsequent PCRs as previously described (*8**,**9*). PCR primers were based either on a multiple alignment of cytochrome b sequences of avian and mammalian species obtained from GenBank or previously published primer sequences cited in Table 2. All DNA templates were initially screened with avian-a and mammalian-a primer pairs, and the sequences were analyzed (Table 2). In some cases, other primer pairs (avian b, mammalian b and c) were additionally used to resolve ambiguous sequences. A Taq PCR Core Kit (Qiagen, Germantown, MD, USA) was used for all PCRs according to the manufacturer's recommendation. A 50-μL reaction volume was prepared with 3 μmL template DNA, 4 μL each primer (0.1–0.5 μmol/L), 5 μL 10× Qiagen PCR Buffer (containing 15 mmol/L MgCl_2_), 1 μL dNTP mix (10 mmol/L each), 0.25 μL Taq DNA polymerase (1.25 U/reaction) and 32.75 μL water. All PCRs were performed with the GeneAmp PCR System 9700 (Applied Biosystems, Foster City, CA, USA) at the ramp speed of 3°C–5°C/s. PCR-amplified products were purified by using QIAquick PCR Purification Kit (Qiagen) and sequenced directly in cycle-sequencing reactions at the Keck Sequencing Facility (Yale University, New Haven, CT, USA) by using the sequencer 3730xl DNA Analyzer (Applied Biosystems). Sequences were annotated by using ChromasPro version 1.22 (Technelysium Pty Ltd., Tewantin, Queensland, Australia) and identified by comparison to the GenBank DNA sequence database (*13*).

**Table 2:** Sequences of primers, length of amplification products, and thermal cycling conditions used in polymerase chain reactions for blood-meal analysis

Primer name	Sequence	Product (bp)	Cycling condition			
			Denaturation	Annealing	Extension	No. cycles
Avian a	GAC TGT GAC AAA ATC CCN TTC CA (f)*	508	94°C (30 s)	60°C (50 s)	72°C (40 s)	36
	GGT CTT CAT CTY HGG YTT ACA AGA C (r)					
Avian b	CCC TCA GAA TGA TAT TTG TCC TCA (f)†	515	95°C (1 min)	58°C (1 min)	72°C (1 min)	35
	CCT CAG AAK GAT ATY TGN CCT CAK GG (r)					
Mammalian a	CGA AGC TTG ATA TGA AAA ACC ATC GTT G (f)	772	94°C (30 s)	55°C (45 s)	72°C (1.5 min)	36
	TGT AGT TRT CWG GGT CHC CTA (r)					
Mammalian b	GCG TAC GCA ATC TTA CGA TCA A (f)	195	95°C (1 min)	54°C (1 min)	72°C (1 min)	32
	CTG GCC TCC AAT TCA TGT GAG (r)					
Mammalian c	CCA TCC AAC ATC TCA GCA TGA TGA AA (f)	395	95°C (1 min)	55°C (1 min)	72°C (1 min)	32
	GCC CCT CAG AAT GAT ATT TGT CCT CA (r)					

*Reference (*39*) †Reference (*40*)

The performance of the molecular based assay was validated by isolating DNA from the blood of a number of known vertebrate species and subjecting it to PCR amplification and DNA sequencing. These species included American robin, American crow, black-capped chickadee, blue jay, button quail, common grackle, eastern tufted titmouse, gray catbird, house sparrow, mourning dove, northern cardinal, sharp-shinned hawk, wood thrush, domestic cat, domestic cow, domestic dog, horse, sheep, white-footed mouse, and white-tailed deer. Similar validation was also conducted with DNA isolated from blood-engorged, laboratory-reared *Aedes aegypti* that fed on guinea pig and button quail. Seasonal changes in the host feeding patterns of *Cx. pipiens* on selected host species were analyzed by χ^2^ analysis for trend by using GraphPad Instat version 3.0 for Windows (GraphPad Software, San Diego, CA, USA).

## Results

Blood-meal sources were successfully identified by DNA sequencing from 204 of 213 *Cx. pipiens*, 30 of 33 *Cx. restuans*, and 100 of 106 *Cx. salinarius*. Of 204 *Cx. pipiens* analyzed, 190 (93.1%) contained avian blood only, 5 (2.5%) mammalian, 1 (0.5%) amphibian, and 8 (3.9%) both avian and mammalian blood. Of 100 *Cx. salinarius* analyzed, 36 (36%) contained avian blood only, 53 (53%) mammalian, and 11 (11%) both avian and mammalian blood. All blood meals identified from *Cx. restuans* were avian-derived.

The composition of avian-derived blood meals is shown in Table 3. We identified 27 species of birds as hosts for *Cx. pipiens*; the most frequently identified species were American robin (40.4 % of avian and 37.7% of total), gray catbird (11.1% and 10.4%), and house sparrow (10.6% and 9.9%). Only 1 American crow–derived blood meal was identified for *Cx. pipiens*. Sixteen bird species were identified as hosts for *Cx. restuans*. American robin (36.7%) was the preferred host for *Cx. restuans*, as it was for *Cx. pipiens*, and no crow-derived blood meals were identified. We identified 13 species of birds as hosts for *Cx. salinarius*. The 2 most common avian species were black-capped chickadee (27.7% of avian and 11.7% of total) and American robin (25.5% and 10.8%). More crow-derived blood meals were identified (8.5% and 3.6%) in this mosquito species.

**Table 3:** Number and percentage of avian blood meals identified from *Culex* mosquitoes collected in Connecticut, 2002–2004

Avian species	Culex pipiens*			*Cx. restuans*		*Cx. salinarius†*		
	No.	% of avian (n = 198)	% of total (n = 212)	No.	% (n = 30)	No.	% of avian (n = 47)	% of total (n = 111)
American robin (*Turdus migratorius*)	80	40.4	37.7	11	36.7	12	25.5	10.8
Gray catbird (*Dumetella carolinensis*)	22	11.1	10.4	2	6.7	–	–	–
House sparrow (*Passer domesticus*)	21	10.6	9.9	–	–	5	10.6	4.5
European starling (*Sturnus vulgaris*)	14	7.1	6.6	1	3.3	1	2.1	0.9
Mourning dove (*Zenaida macroura*)	13	6.6	6.1	2	6.7	3	6.4	2.7
Black-capped chickadee (*Poecile atricapilla*)	9	4.5	4.2	1	3.3	13	27.7	11.7
Common grackle (*Quiscalus quiscula*)	8	4.0	3.8	2	6.7	–	–	–
Wild turkey (*Meleagris gallopavo*)	6	3.0	2.8	1	3.3	–	–	–
Northern cardinal (*Cardinalis cardinalis*)	4	2.0	1.9	2	6.7	–	–	–
House finch (*Carpodacus mexicanus*)	3	1.5	1.4	–	–	1	2.1	0.9
Barn swallow (*Hirundo rustica*)	2	1.0	0.9	–	–	–	–	–
American crow (*Corvus brachyrhynchos*)	1	0.5	0.5	–	–	4	8.5	3.6
Sharp-shinned hawk (*Accipiter striatus*)	1	0.5	0.5	1	3.3	1	2.1	0.9
Brown-headed cowbird (*Molothrus ater*)	1	0.5	0.5	1	3.3	1	2.1	0.9
Canada goose (*Branta canadensis*)	1	0.5	0.5	1	3.3	1	2.1	0.9
Wood thrush (*Hylocichla mustelina*)	1	0.5	0.5	1	3.3	–	–	–
Red-winged blackbird (*Agelaius phoeniceus*)	1	0.5	0.5	–	–	–	–	–
Cedar waxwing (*Bombycilla cedrorum*)	1	0.5	0.5	–	–	–	–	–
Red-tailed hawk (*Buteo jamaicensis*)	1	0.5	0.5	–	–	–	–	–
Green heron (*Butorides virescens*)	1	0.5	0.5	–	–	–	–	–
Rock dove (*Columba livia*)	1	0.5	0.5	–	–	–	–	–
Song sparrow (*Melospiza melodia*)	1	0.5	0.5	–	–	–	–	–
Indigo bunting (*Passerina cyanea*)	1	0.5	0.5	–	–	–	–	–
House wren (*Troglodytes aedon*)	1	0.5	0.5	–	–	–	–	–
Willow flycatcher (*Empidonax traillii*)	1	0.5	0.5	–	–	–	–	–
Black-and-white warbler (*Mniotilta varia*)	1	0.5	0.5	–	–	–	–	–
Northern waterthrush (*Seiurus noveboracensis*)	1	0.5	0.5	–	–	–	–	–
Wood duck (*Aix sponsa*)	–	–	–	–	–	2	4.3	1.8
Prairie warbler (*Dendroica discolor*)	–	–	–	1	3.3	–	–	–
Mallard (*Anas platyrhynchos*)	–	–	–	–	–	1	2.1	0.9
Northern oriole (*Icterus galbula*)	–	–	–	1	3.3	–	–	–
Black-crowned night heron (*Nyctiocorax nyctiocorax*)	–	–	–	–	–	2	4.3	1.8
Rose-breasted grosbeak (*Pheucticus ludovicianus*)	–	–	–	1	3.3	–	–	–
Blue-headed vireo (*Vireo solitarius*)	–	–	–	1	3.3	–	–	–

*Includes 8 specimens from which double-blood meals were identified. †Includes 11 specimens from which double-blood meals were identified.

A seasonal shift from American robins to other avian species was noted with *Cx. pipiens* (Table 4). The χ^2^ test for linear trend showed that the proportion of American robin–derived blood meals decreased from June until October (p<0.0001). In June, 62.4% of all avian-derived blood meals were obtained from American robins, and this percentage declined to 26.7% in July and 38.9% in August. By September, 25.7% of the avian-derived blood meals were obtained from gray catbirds and 20.0% from mourning doves, while none was identified as being from American robins.

**Table 4:** Monthly prevalence of avian-derived blood meals in *Culex pipiens*

Month	Total avian	American robin, n (%)	Gray catbird, n (%)	House sparrow, n (%)	Mourning dove, n (%)	European starling, n (%)	Other species, n (%)
June	93	58 (62.4)	2 (2.2)	4 (4.3)	–	12 (12.9)	17 (18.3)
July	30	8 (26.7)	2 (6.7)	9 (30.0)	3 (10.0)	2 (6.7)	6 (20.0)
August	36	14 (38.9)	6 (16.7)	4 (11.1)	3 (8.3)	–	9 (25.0)
September	35	–	9 (25.7)	3 (8.6)	7 (20.0)	–	16 (45.7)
October	4	–	3 (75.0)	1 (25.0)	–	–	–
Total	198	80	22	21	13	14	48

An analysis of the mammalian blood meal sources for *Cx. pipiens* and *Cx. salinarius* is shown in Table 5. We identified 10 host species for *Cx. salinarius* and 7 for *Cx. pipiens*. White-tailed deer (*Odocoileus virginianus*) was the most frequently identified host for *Cx. salinarius* (67.2% of mammalian and 38.7% of total). Human-derived blood meals were identified from 2 *Cx. salinarius* and 1 *Cx. pipiens*.

**Table 5:** Number and percentage of mammalian blood meals taken by *Culex* mosquitoes collected in Connecticut, 2002–2004

Species	Culex pipiens*			*Cx. salinarius†*		
	No.	% of mammal (n = 13)	% of total (n = 212)	No.	% of mammal (n = 64)	% of total (n = 111)
White-tailed deer (*Odocoileus virginianus*)	4	30.8	1.9	43	67.2	38.7
Gray squirrel (*Sciurus carolinensis*)	3	23.1	1.4	–	–	–
Northern raccoon (*Procyon lotor*)	2	15.4	0.9	2	3.1	1.8
Human (*Homo sapiens*)	1	7.7	0.5	2	3.1	1.8
Virginia opossum (*Didelphis virginiana*)	1	7.7	0.5	4	6.2	3.6
Dog (*Canis familiaris*)	–	–	–	4	6.2	3.6
Cat (*Felis catus*)	1	7.7	0.5	3	4.7	2.7
Eastern cottontail (*Sylvialagus floridanus*)	1	7.7	0.5	3	4.7	2.7
Horse (*Equus caballus*)	–	–	–	1	1.6	0.9
Striped skunk (*Memphitis memphitis*)	–	–	–	1	1.6	0.9
Brown rat (*Rattus norvegicus*)	–	–	–	1	1.6	0.9

*Includes 8 specimens from which double-blood meals were also identified. †Includes 11 specimens from which double-blood meals were also identified.

## Discussion

Our analysis on the blood-feeding behavior of *Culex* mosquitoes provides insight into their relative roles as enzootic and epidemic vectors of WNV in this region of the northeastern United States. We found that *Cx. pipiens* and *Cx. restuans* predominantly feed on avian hosts and focus their feeding activity on several key bird species that can support WNV transmission, in particular, American robins, gray catbirds, and house sparrows. By contrast, we found that *Cx. salinarius* feeds more opportunistically than *Cx. pipiens* and *Cx. restuans* and includes a relatively high proportion of mosquitoes with mixed blood meals from both avian and mammalian sources. This finding suggests that *Cx. salinarius* serves as a bridge vector by transferring WNV from viremic birds to mammalian hosts.

The preponderance of WNV isolations obtained from *Cx. pipiens* in surveillance activities conducted over the last 6 years (*6**,**10**,**14**,**15*) clearly incriminates this species as the predominant mosquito vector in this region. However, while enzootic transmission to birds is strongly supported by a number of host-preference studies on regional populations (*8**,**9**,**16**–**18*), no consensus has been reached on the role of *Cx. pipiens* in epidemic transmission of WNV to humans in the northeastern United States. Apperson et al. (*9*) recently identified mammalian-derived blood meals in 38% of blood-fed *Cx. pipiens*, 10.8% of which were human-derived (≈2.5% overall), collected from New Jersey. This finding led these researchers to conclude that *Cx. pipiens* was likely an epidemic vector in that region. This interpretation was viewed as consistent with the incidence of human cases in 3 densely populated urban areas of Connecticut in 2002, where most viral isolations (78%) were from *Cx. pipiens* (*6*). Kilpatrick et al. (*7*), integrating WNV testing data from New York from 2000 to 2003 with information on mosquito abundance, infection prevalence, vector competence, and biting behavior, further suggested that *Cx. pipiens* and *Cx. restuans* were responsible for up to 80% of human infections in that region. However, the validity of their conclusions was based on the identification of mammalian-derived blood in ≈19% of these 2 species and the assumption that humans were also included. Our analysis of blood meals from wild-caught female *Cx. pipiens* from established WNV foci in Connecticut is inconsistent with this supposition, as this species shows a strong tendency for avian blood and little inclination for mammalian hosts, including humans. We, therefore, conclude that while *Cx. pipiens* may occasionally feed on humans, it may not be the predominant vector of WNV to humans in our region of the northeastern United States. This finding is compatible with the lack of any mammalian-derived blood meals in blooded *Cx. pipiens* collected from suburban locales in nearby Westchester County, New York (*9*), but contrasts sharply with a recent study conducted in Delaware, where 69% of the blood meals taken by *Cx. pipiens* were from large mammals (*19*), which suggests a difference in host preference from more southern regions of its range.

Examination of the blood-fed mosquitoes in the present study showed an exclusively ornithophilic nature of *Cx. restuans*; all analyzed blood meals were from avian species. These findings were consistent with prior host preference studies (*8**,**9**,**16**,**20*) and strongly support the view that this predominant "early season" species is most likely involved in initiation and amplification of WNV transmission among wild birds and rarely, if ever, feeds on humans in this region. This finding differs from a recent blood-meal analysis by Gingrich and Williams (*19*), who found that a limited number (n = 9) of *Cx. restuans* from Delaware were highly mammalophilic (9:1 mammal-to-bird ratio). However, they concluded that this species was still primarily an enzootic vector since they never collected it in human landing collections.

Our findings regarding the blood-feeding patterns of *Cx. salinarius* reinforce those of previous studies (*18**,**20**–**25*) and indicate that this species feeds indiscriminately on both birds and mammals, including humans. By using separate PCR primer pairs for different vertebrate classes, we find that 11% of *Cx. salinarius* acquired blood meals from both avian and mammalian sources, versus ≈4% for *Cx. pipiens*. We cannot say whether all or most of these double-source blood meals represent multiple feeding episodes during the same gonotrophic cycle or the detection of residual DNA from a prior egg-laying cycle. However, mixed-source blood meals have been reported for a number of *Culex* species by using different methods for blood-meal identification (*8**,**16*). Regardless, our findings indicate that a relatively large fraction of the *Cx. salinarius* population readily feeds on both birds and mammals, which is a necessary condition for epidemic transmission to humans. The opportunistic feeding pattern of *Cx. salinarius*, in conjunction with its physiologic competence to transmit WNV (*26*), high infection rates in nature (*10**,**14**,**15*), and seasonal distribution that overlaps with human cases (*6*), all indicate that this species is a bridge vector of WNV to humans in the northeastern United States.

White-tailed deer were the single most important source of blood for *Cx. salinarius* in our study, which supports similar findings from New Jersey (*9**,**18*). The apparent affinity of *Cx. salinarius* for deer over other mammalian hosts is likely a function of deer's availability, as they are the most abundant large mammals in the region after humans. The role of deer in the ecology and transmission dynamics of WNV is unknown. Seroprevalence of WNV antibodies was 0%–6% among hunter-killed deer from New Jersey in 2001 (*27*), which suggests infrequent exposure to WNV relative to avian hosts, but frequency of exposure is still greater than that in humans (*28*). Widespread abundance of deer could be zooprophylactic by diverting feeding from avian amplifying hosts to deer. This possibility merits further study.

Several avian hosts are highly susceptible to WNV infection and can support viremia sufficient to infect culicine vectors. Reservoir competence values expressed as the duration and magnitude of infectious-level viremia were evaluated for 25 bird species and shown to be highest for passerine birds, including the blue jay, common grackle, house finch, American crow, house sparrow, and American robin (*29*). Field data further implicate a few species as reservoir hosts in northeastern United States on the basis of their exposure to WNV. When abundance and seroprevalence data were combined, house sparrows were estimated to be the most commonly infected bird species in New York City (*30**,**31*). These findings, combined with reservoir competence data, suggest that house sparrows are amplifying hosts in urban locales; however, other resident bird species, such as the northern cardinal, house finch, and gray catbird, were also frequently exposed to WNV (*30*). We show that in Connecticut, *Cx. pipiens* and *Cx. restuans* acquire blood meals predominately from American robins, implicating this species as a reservoir host for WNV. American robins are moderately competent and develop infectious-level viremia for a duration of ≈3 days (*29*). This species is most abundant in Connecticut from early spring to midsummer (*32*). Therefore, they may support more early- to mid-season (June–August) amplification of the virus. Our findings of a seasonal shift in *Cx. pipiens* from American robins to other avian species support this hypothesis.

We found that *Cx. pipiens* and *Cx. restuans* rarely fed upon American crows, despite their abundance (*32*) and high death rate from WNV infection throughout the region (*33**–**35*). Similar findings were reported from *Cx. pipiens*–complex mosquitoes collected from New York (*8*) and New Jersey (*9*). This finding suggests that American crows may also acquire WNV through other means in addition to mosquito transmission. American crows are susceptible to WNV infection by oral ingestion of the virus in aqueous solution and by eating infected bird carcasses (*29*). These birds are aggressive nest raiders and, therefore, could also acquire WNV infection by eating infected nestling birds. Transmission could also occur directly from bird to bird, as has been demonstrated in laboratory settings for this and other species (*29**,**36**,**37*). Our findings indicate that American crows may not be the primary amplifying hosts for infecting *Culex* mosquitoes with WNV in this region of the northeastern United States. Alternatively, we find that other common birds, including American robins, gray catbirds, and house sparrows, may play a greater role in supporting enzootic transmission.

Our PCR-based method took advantage of the conservation and diversity of mitochondrial sequences in identifying the source of vertebrate blood from mosquitoes. Mitochondrial DNA is a useful marker in phylogenetic studies and molecular systematics because of its maternal inheritance, haploid nature, and rapid rate of evolution (*38*). The cytochrome b gene, in particular, has successfully been used to identify taxonomic groups to the subspecies level and these sequences are publicly accessible from a wide array of different bird and mammal species in the GenBank database. By sequencing portions of the cytochrome b gene, we unambiguously identified the blood-meal source to the species level, which represents an improvement in sensitivity and specificity over earlier analyses.
